# Supplementary material for: Deciphering the history of ERK activity from fixed-cell immunofluorescence measurements
Source: Nat Commun. 2025 May 21;16:4721. doi: 10.1038/s41467-025-58348-7 (PMC12095524; doi:10.1038/s41467-025-58348-7)
Supplement: Supplementary file 1 — Supplementary Information [file 41467_2025_58348_MOESM1_ESM.pdf]

Supplementary Figure 1

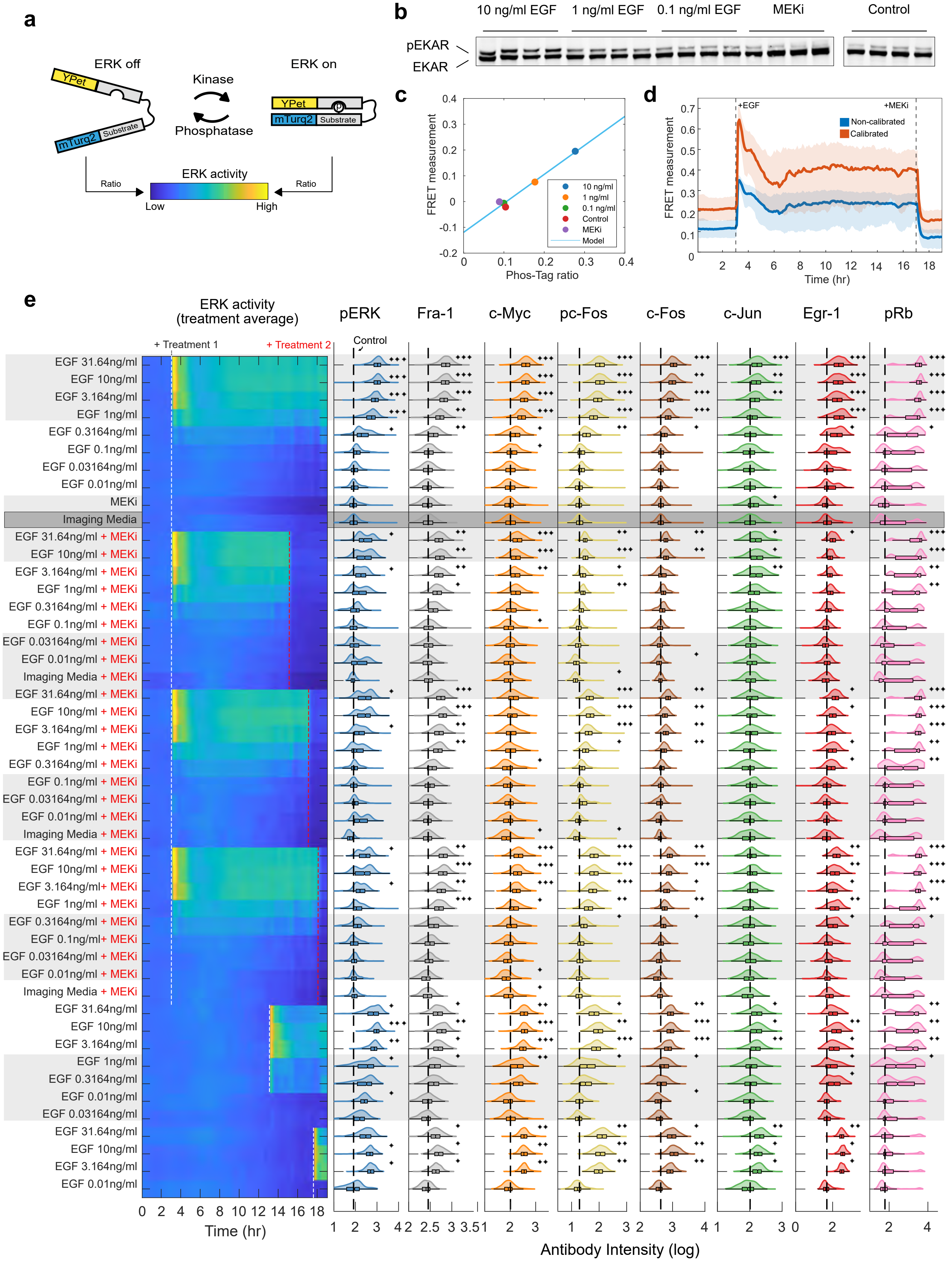

Supplementary Figure 1: Live cell measurements with a calibrated ERK reporter followed by immunofluorescence. a Schematic of EKAR3.1 FRET-based reporter. When ERK is inactive, mTurquoise2 and Ypet are distanced from each other. Active ERK binds the reporter substrate and induces a conformational change, bridging the two fluorescent proteins together. This causes a change in the ratio of mTurquoise2 and Ypet fluorescence intensities**. b** Phos-Tag immunoblot for phospho-EKAR3.1 under 4 conditions that span the full range of ERK activity levels. Samples treated with EGF for 15 minutes, or MEKi for 2 hours. $n_{well\ replicates} = 4$ for each treatment. **c** Quantified ratio of the phosphorylated EKAR3.1 over total EKAR3.1 immunoblot intensities (x-axis). Y-axis represents the average live-cell FRET measurement in all cells within each treatment. FRET measurements were calculated at 15 minutes after EGF treatment, or 2 hours after MEKi. Each point represents the average of the 4 replicates. Model indicates the line of best fit. **d** Slope and intercept of the Phos-Tag model were used to calibrate the live-cell FRET measurements. **e** Left: Treatment average responses of EKAR biosensor data. Right: Histogram and box plot showing immunofluorescence quantifications for each condition corresponding to the biosensor data. Box plot indicates median, quartiles, and range of the data. Dashed line indicates the median of the control (imaging media). Variance-corrected t-tests were conducted by comparing each EGF treated condition to vehicle control (imaging media) ($n_{replicates} = 3$). * p-val < 0.05, ** p-val < 0.005, *** p-val < 0.0005.

Supplementary Figure 2

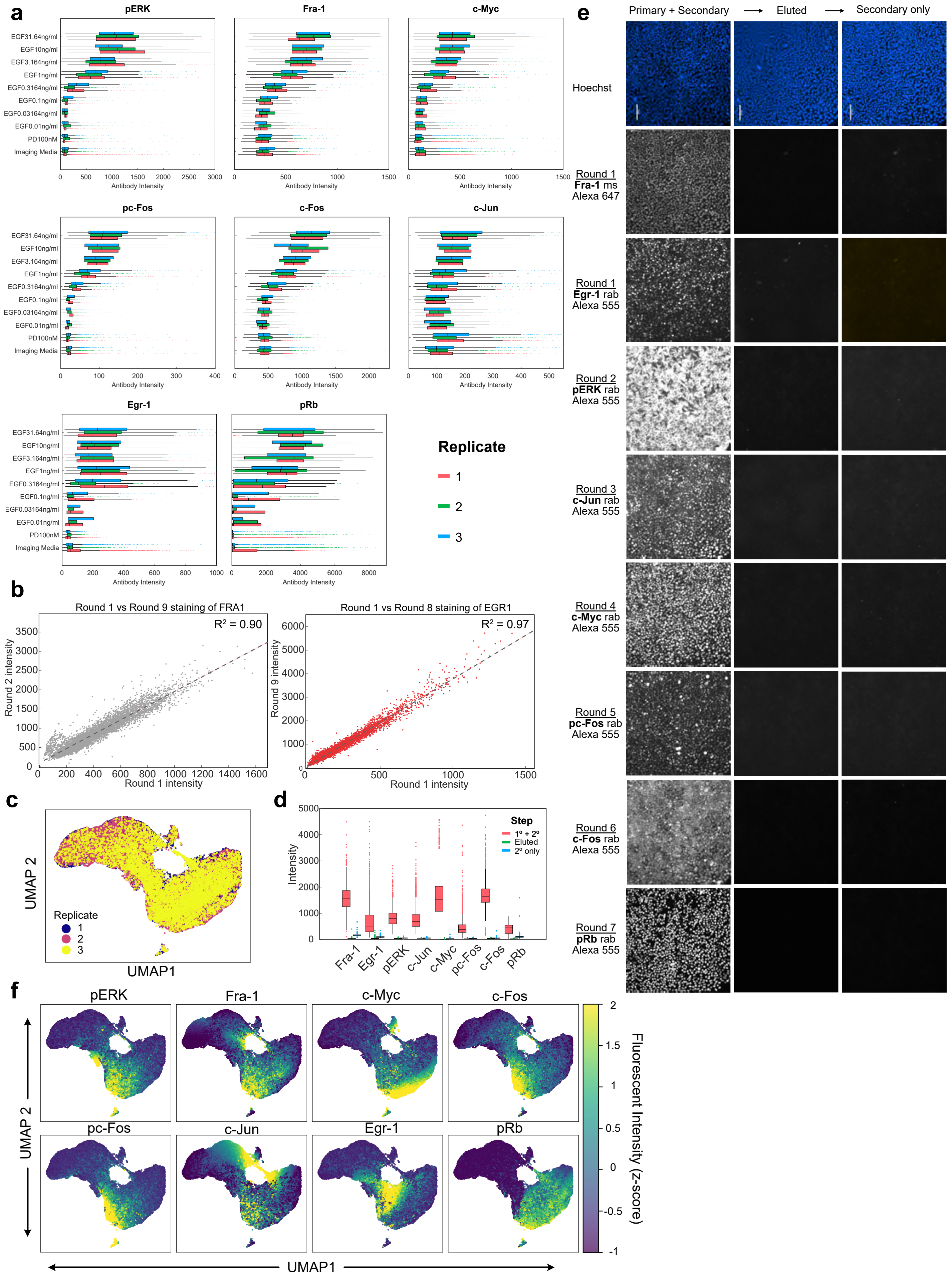

Supplementary Figure 2: Batch effect correction and cyclic immunofluorescence protocol validation. a Box plots showing immunofluorescence quantifications for each condition in each replicate experiment. Box plot indicates median, quartiles, and range of the data. Dots indicate outliers. $n_{replicates}$ = 3 **b** Scatter plot of Fra-1 (left) and Egr-1 (right) intensity in the first round of staining vs. the ninth round from replicate plate 1. Data includes cells treated with EGF (all doses), imaging media, or MEKi. $n_{well\ replicates}$ = 2 for each treatment**. c** Uniform Manifold Projection (UMAP) colored by replicate plate, created with nuclear measurements of 8 ETGs. **d** Quantification of nuclear pixel intensities of cells in e. Box plot indicates median, quartiles, and range of the data. Dots indicate outliers. **e** Images of cyclic immunofluorescence after each round of staining. Cells were incubated with primary and then secondary antibodies, then eluted and re-incubated with secondary antibody only to ensure proper elution of the primary antibody. Anti-Egr-1 (rabbit, Rab) and anti Fra-1 (mouse, Ms) antibodies were both incubated together in round 1. Cells shown were treated with 31.64 ng/ml EGF. $n_{well\ replicates}$ = 1. Five other wells treated with lower concentrations of EGF were also imaged and validated for proper elution (data not shown). The panel shows registered images of the same image field at the same magnification, with the scale bar for all images shown in the Hoechst channel. **f** UMAP projections colored by immunofluorescence intensity of indicated stain.

Supplementary Figure 3

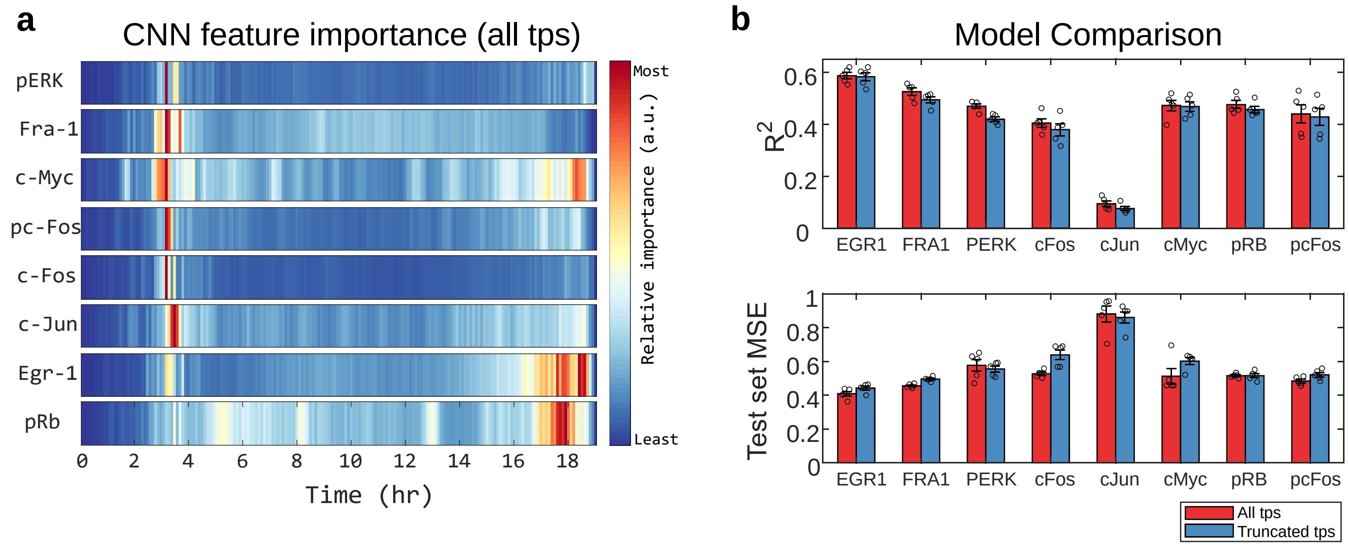

Supplementary Figure 3: CNN feature importance is overshadowed by initial response when all timepoints are included. a Convolutional neural net feature importance of each timepoint in predicting levels of each ETG, using the entire live-cell time course for training. Note that time points immediately following growth factor addition (just prior to 4 hr) dominate the feature importance, even though activity at this time point is unlikely to provide direct information on the ETG status at the end of the experiment. It is likely that this outsized importance reflects the model learning the ERK pathway responsiveness of each cell from the initial response to growth factor. As in Fig. 3d, color map represents relative values within each row. **b** Comparison of CNNs trained on 190 timepoints (19 hr, panel **a**) or 150 time points (15hr, as shown in main Fig. 3d). Top: Bar plot of R^2 value for predicting each ETG using k-fold cross-validation (k=5). For each ETG, data were partitioned into 5 groups. Within each k-fold, a training, test, and final set were created. Bar represents the average final set R2 value across all 5 groups. Error bars (Standard error) were calculated by dividing the standard deviation of R2 values for each ETG by the square root of five. Bottom: Test set mean squared error values for each ETG. Bar height and error bars were calculated as described above.

Supplementary Figure 4

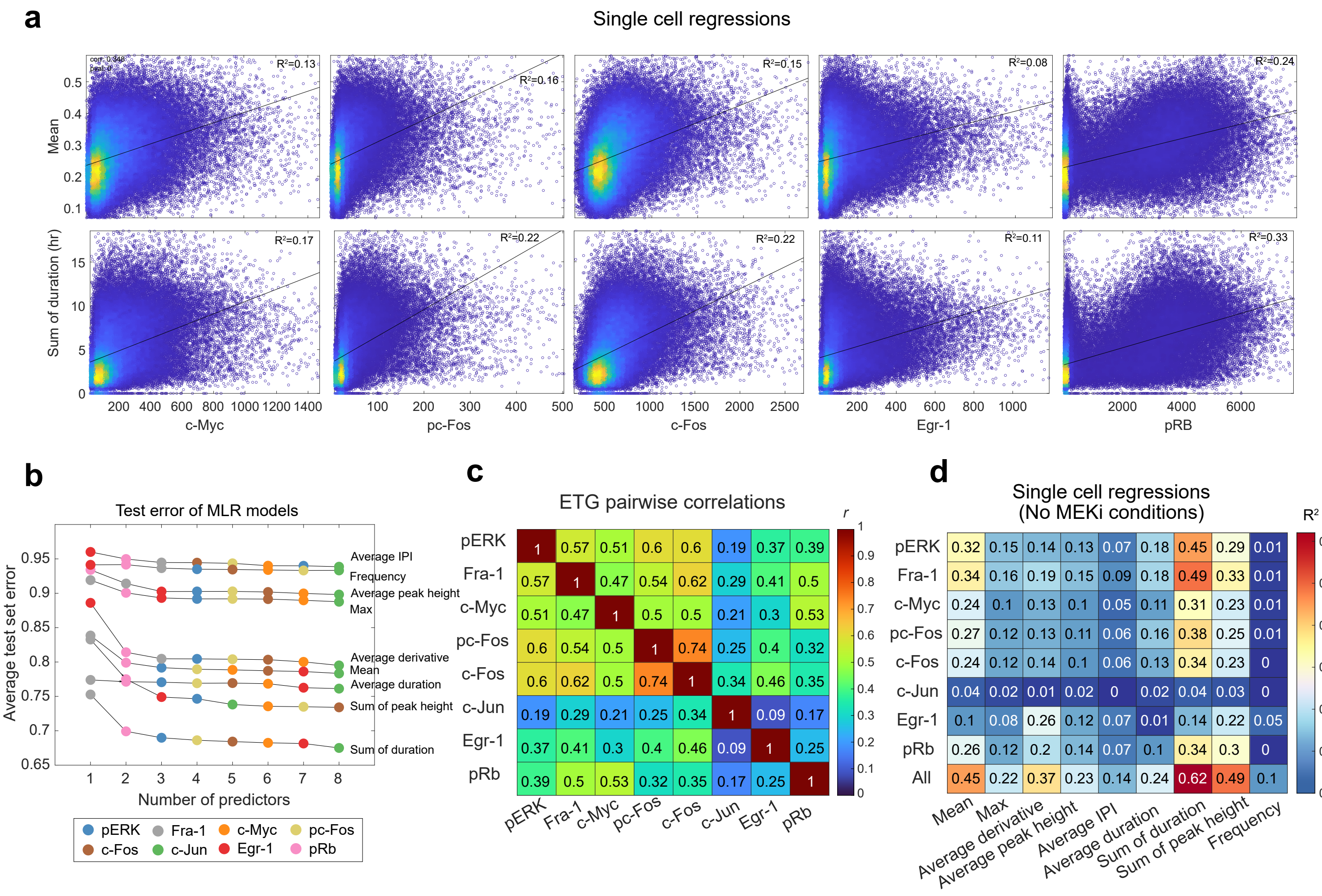

Supplementary Figure 4: Regression modeling of ERK and ETGs. a Scatter plots and line of best fit for ETGs and ERK features. Color indicates relative density of data. Outliers not shown. **b** Test error (RMSE) of MLR models where additional predictors were added at each step. **c** Pearson correlation between each ETG. **d** Single variable regression models using single-cell data, cells treated with MEKi were removed from this analysis.

Supplementary Figure 5

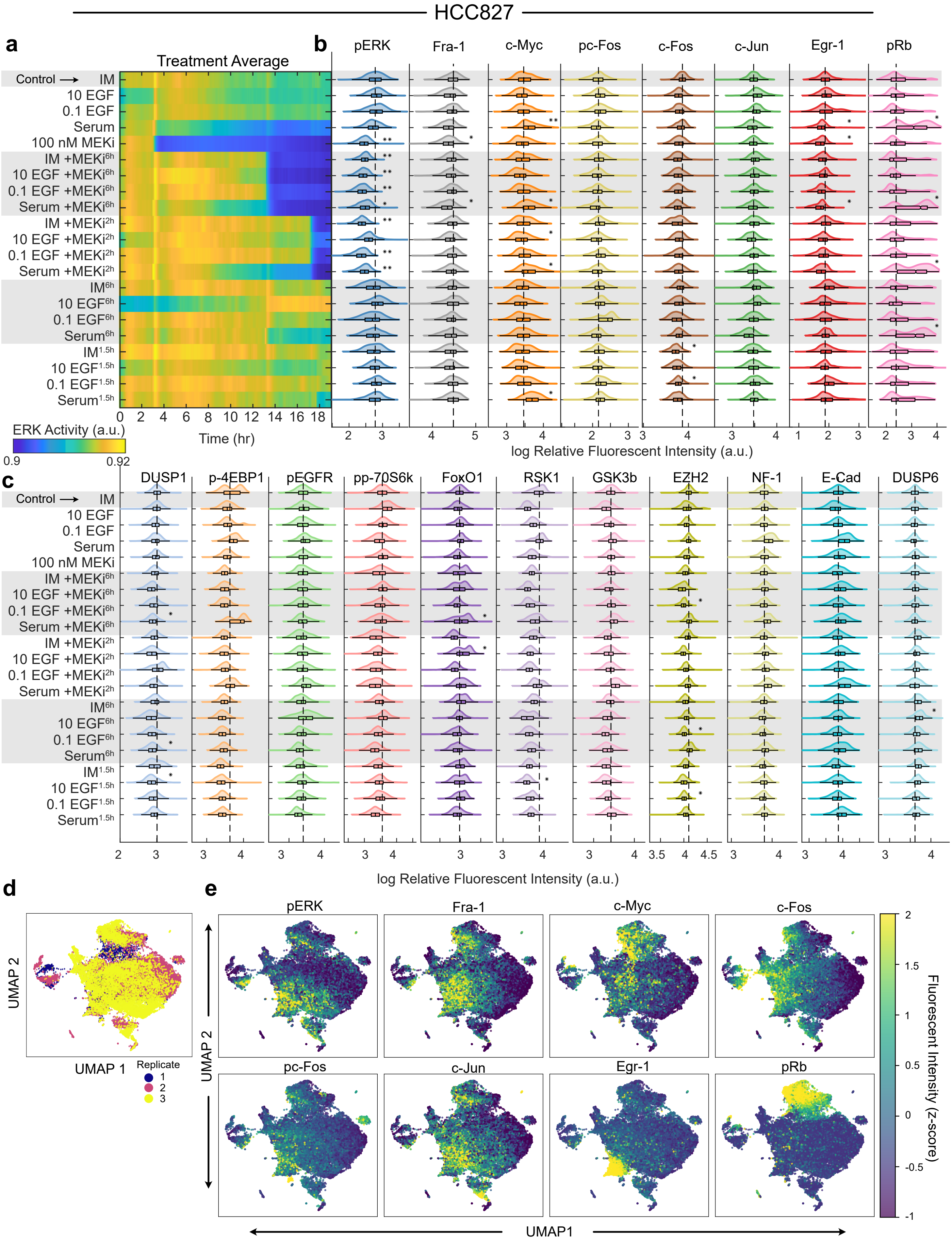

Supplementary Figure 5: Dataset summary for HCC827 cell line. a Left: Condition average responses of EKAR biosensor data. All treatments added at the 3 hr timepoint unless otherwise indicated by superscripts. EGF dose are ng/ml (units removed for brevity). Superscripts (e.g. $^{1.5h}$) denote time of addition, prior to fixation. MEKi: PD0325901 (single perturbation wells: 100nM, dual perturbation wells: 200nM). Serum: 10% FBS. **b-c** Histogram and box plot showing immunofluorescence (IF) quantifications for each treatment corresponding to the biosensor data. Box plot indicates median, quartiles, and range of the data. Dashed line indicates the median of the control, imaging media (IM). Variance-corrected t-tests were conducted by comparing each EGF treated condition to IM ($n_{replicates} = 3$). * p-val < 0.05, ** p-val < 0.005, *** p-val < 0.0005. **d** UMAP of immunofluorescence data, colored by replicate plate of origin. Batch effect corrected data was then z-scored before running UMAP **e** UMAP colored by immunofluorescence intensity of indicated stain.

Supplementary Figure 6

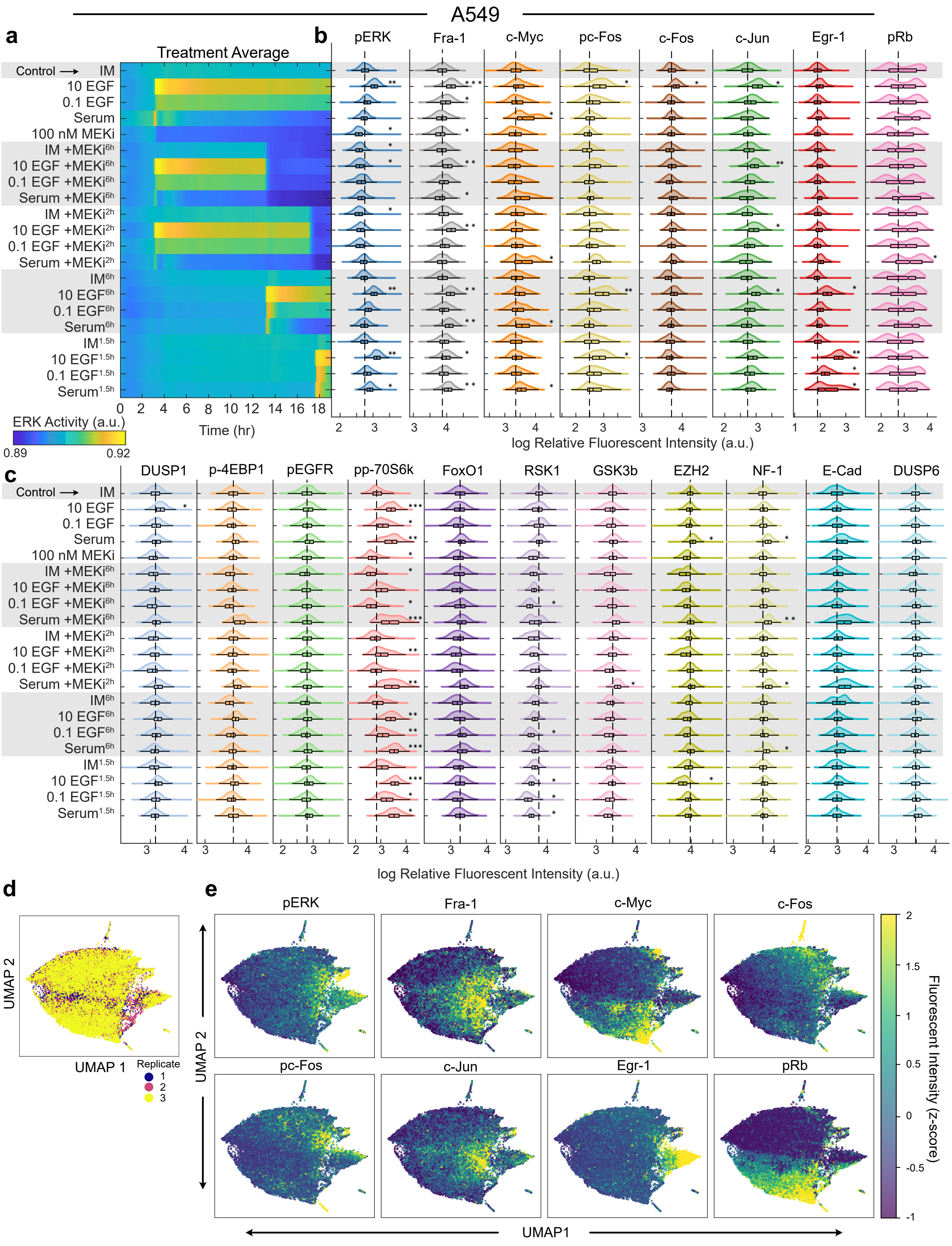

Supplementary Figure 6: Dataset summary for A549 cell line. a Left: Condition average responses of EKAR biosensor data. All treatments added at the 3 hr timepoint unless otherwise indicated by superscripts. EGF dose are ng/ml (units removed for brevity). Superscripts (e.g. $^{1.5h}$) denote time of addition, prior to fixation. MEKi: 100 nM PD0325901. Serum: 10% FBS. **b-c** Histogram and box plot showing immunofluorescence (IF) quantifications for each treatment corresponding to the biosensor data. Box plot indicates median, quartiles, and range of the data. Dashed line indicates the median of the control, imaging media (IM). Variance-corrected t-tests were conducted by comparing each EGF treated condition to IM ($n_{replicates} = 3$). * p-val < 0.05, ** p-val < 0.005, *** p-val < 0.0005. **d** UMAP of immunofluorescence data, colored by replicate plate of origin. Batch effect corrected data was then z-scored before running UMAP **e** UMAP colored by immunofluorescence intensity of indicated stain.

Supplementary Figure 7

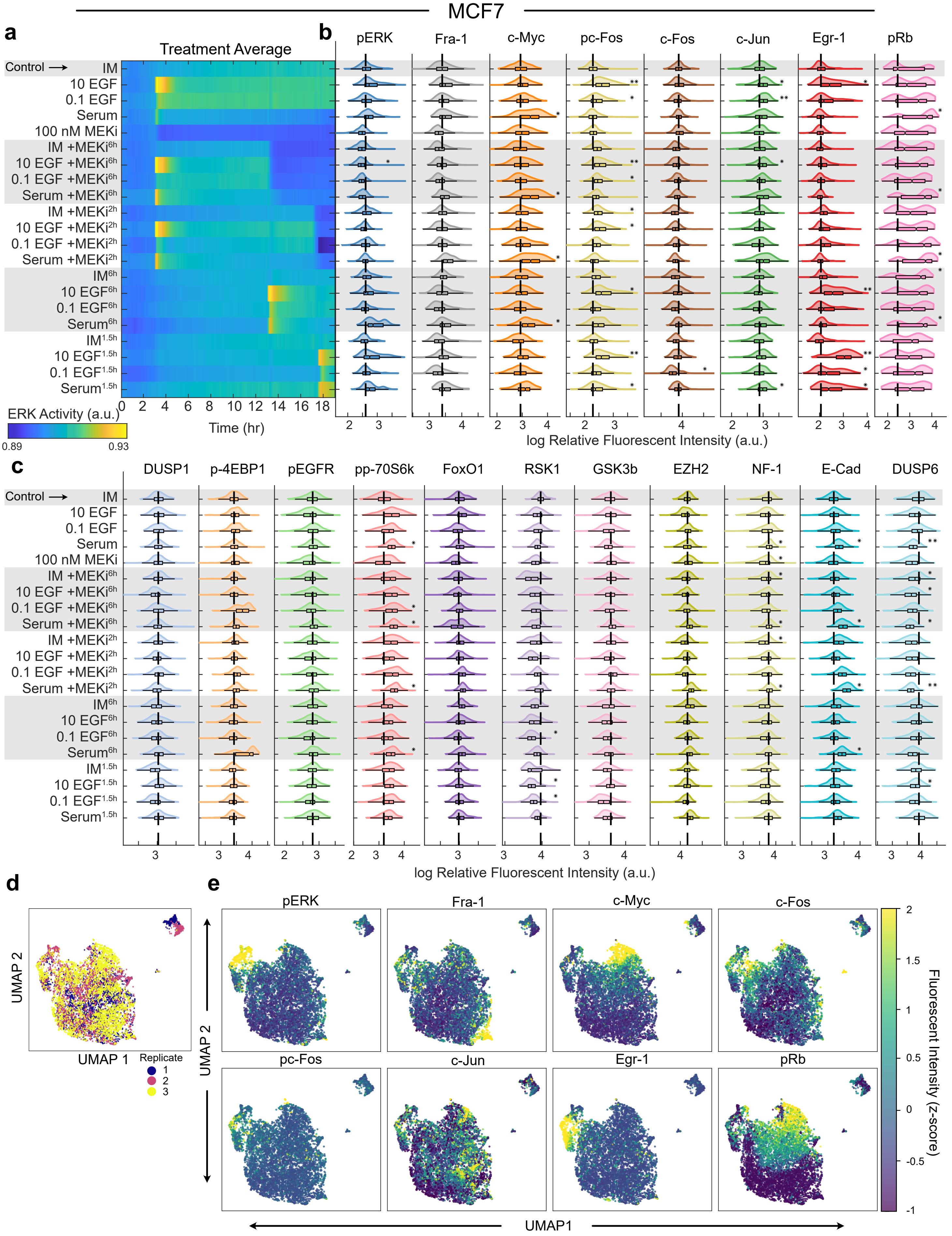

Supplementary Figure 7: Dataset summary for MCF7 cell line. a Left: Condition average responses of EKAR biosensor data. All treatments added at the 3 hr timepoint unless otherwise indicated by superscripts. EGF dose are ng/ml (units removed for brevity). Superscripts (e.g. $^{1.5h}$) denote time of addition, prior to fixation. MEKi: 100 nM PD0325901. Serum: 10% FBS. **b-c** Histogram and box plot showing immunofluorescence (IF) quantifications for each treatment corresponding to the biosensor data. Box plot indicates median, quartiles, and range of the data. Dashed line indicates the median of the control, imaging media (IM). Variance-corrected t-tests were conducted by comparing each EGF treated condition to IM ($n_{replicates} = 3$). * p-val < 0.05, ** p-val < 0.005, *** p-val < 0.0005. **d** UMAP of immunofluorescence data, colored by replicate plate of origin. Batch effect corrected data was then z-scored before running UMAP **e** UMAP colored by immunofluorescence intensity of indicated stain.

Supplementary Figure 8

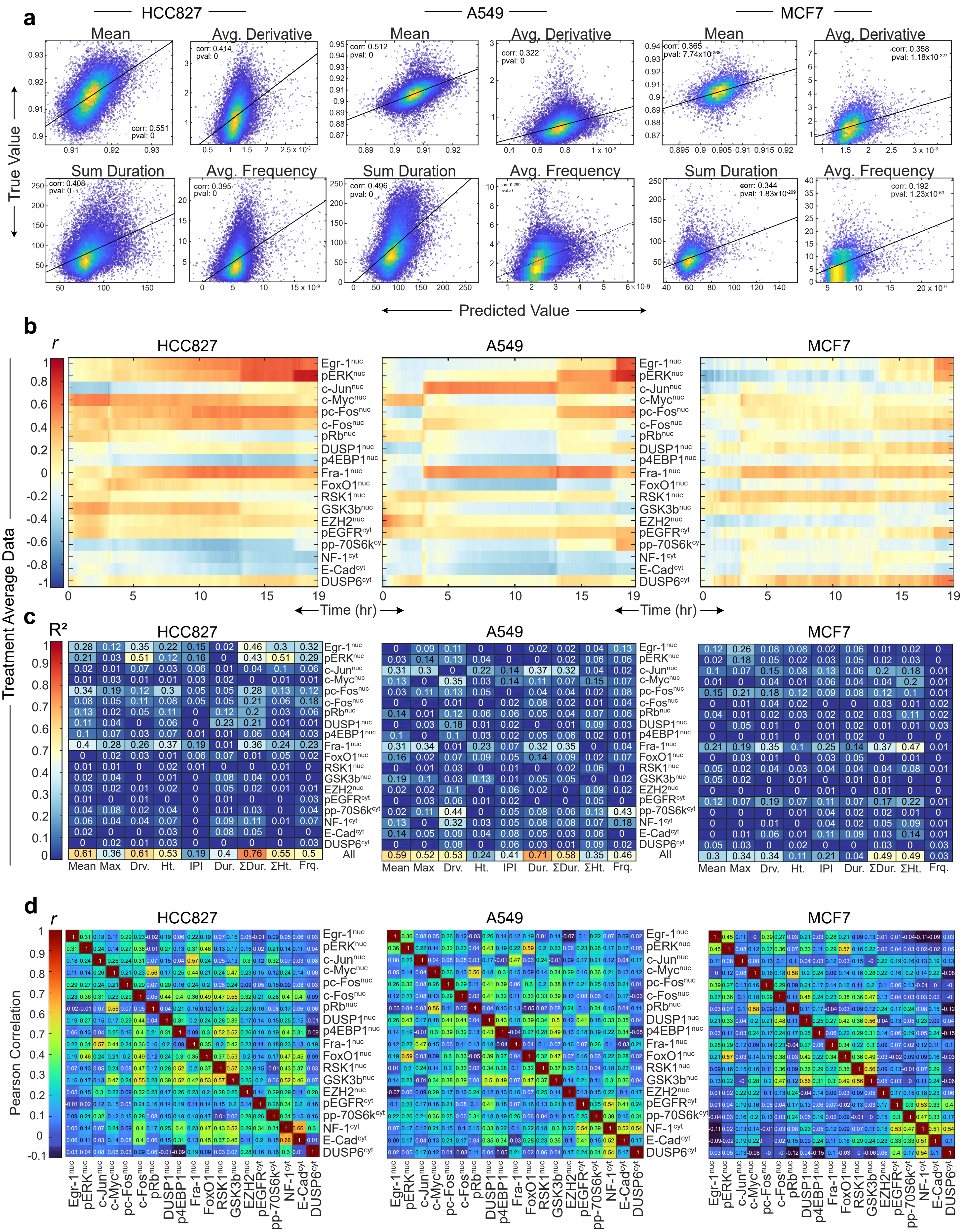

Supplementary Figure 8: Cancer cell types display deficiencies in processing ERK dynamics. a Selected scatter plots of single-cell multiple regression models showing line of best fit. Color indicates relative density of the data. Outlines not shown **b** Pearson correlation (r) between treatment average protein measurements and the average EKAR FRET measurement at each timepoint from the live-cell experiment. **c** Average values were calculated for all cells with the same treatment. These values were then used to fit cross validated regression models that predict each ERK feature using antibody measurements. "All" indicates multiple regression models using all proteins as predictors. Drv: Average Derivative. Ht: Average Pulse Height. IPI: Average Inter-peak Interval. Dur: Average Duration. ΣDur: Sum of Pulse Duration. ΣDur: Sum of Pulse Height. Frq: Average Frequency. **d** Pearson correlation between each antibody measurement within single cells.

Supplementary Figure 9

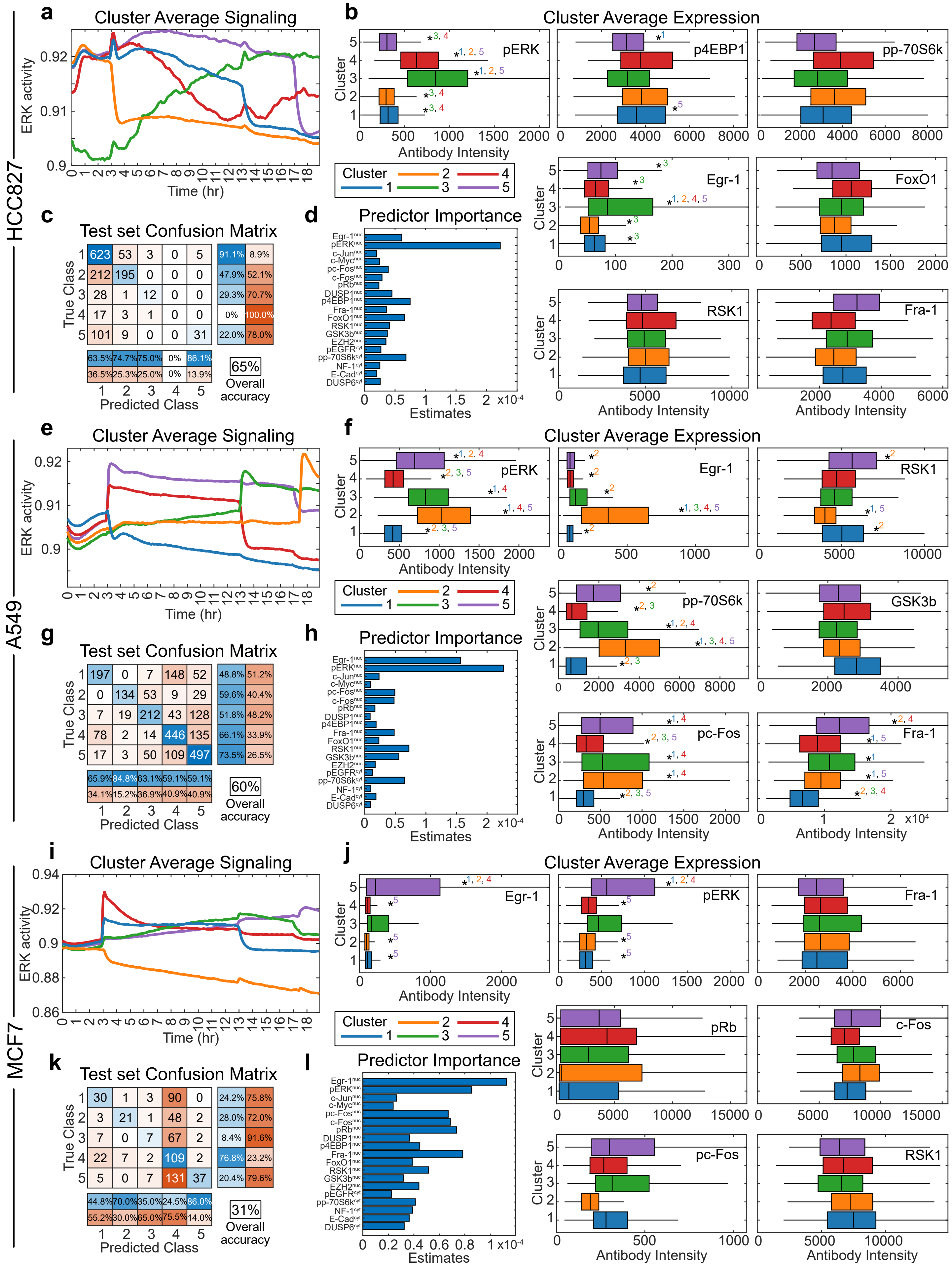

Supplementary Figure 9: Prototypes of ERK signaling are captured by ETGs in HCC827, A549, and MCF7 cells. a Average ERK activity in each class identified by k-means (k = 5) clustering of EKAR time series data in HCC827 single-cells. **b** Box plot showing median, quartiles, and range of ETG intensity in each class identified in **a**. One way ANOVA test was conducted to compare the means of each group to each other. All comparisons are not significant unless otherwise indicated. * pval < 0.05 compared to indicated group. **c** Confusion matrix indicating correct and incorrect predictions for each class identified in **a**. AdaBoostM2 algorithm was trained to predict the cluster ID of each cell using its ETG measurements as predictors. 10-fold cross-validation was conducted to retrieve the best test-set model. **d** Predictor importance estimates of each ETG in the model in **c**. **e-l** Repeated analysis, as a-d, in A549 and MCF7 cells.

Supplementary Figure 10

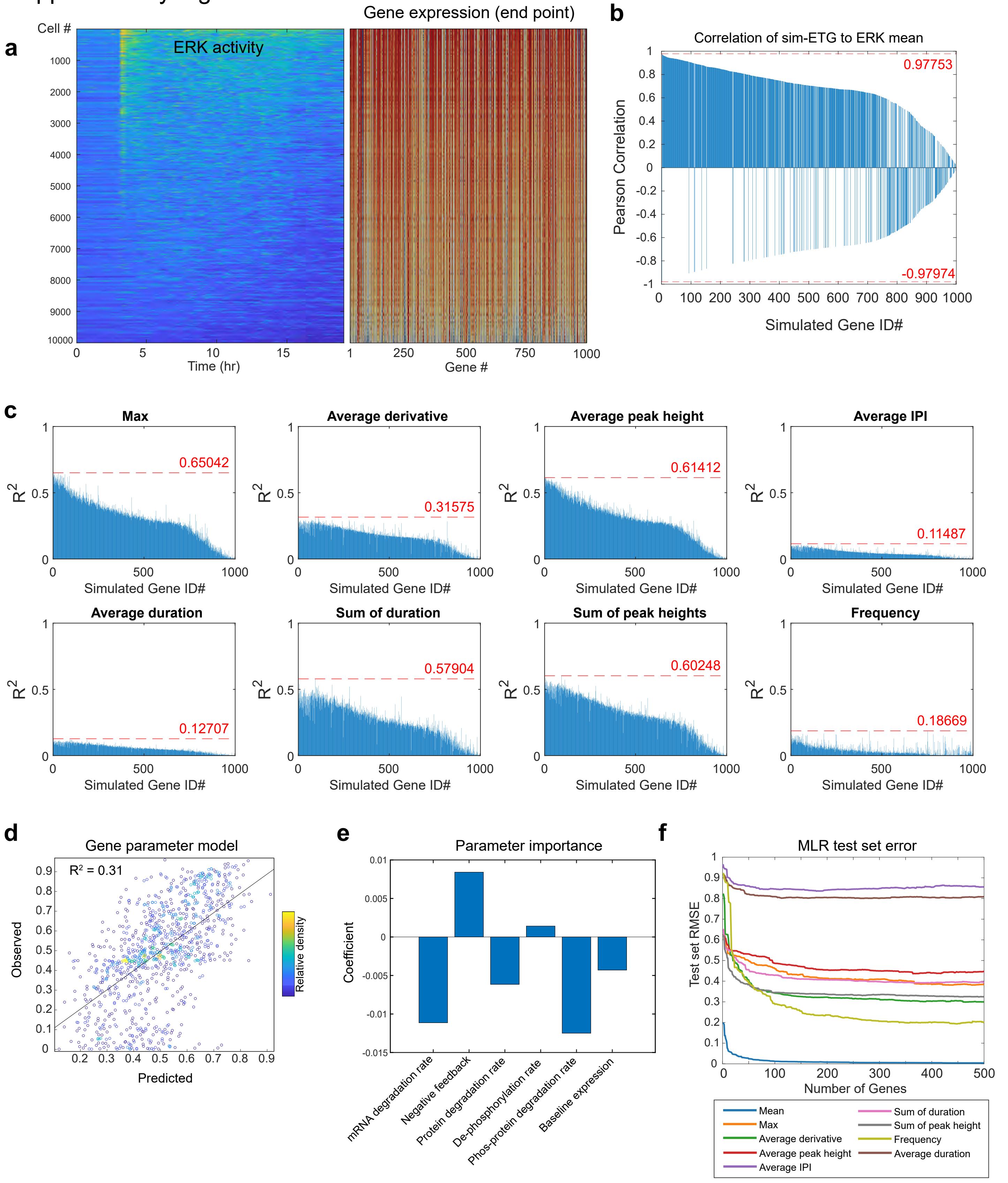

Supplementary Figure 10: Ordinary differential equation modeling. a Left: single-cell ERK activity heatmap sorted by the mean of each cell (highest mean at the top). Right: Corresponding sim-ETG end point values. Color represents the relative expression within each column. n_{cells} = 10,000. n_{genes} = 1,000**.** **b** Pearson correlation between mean ERK activity and end-point gene expression. **c** R^2 of single variable models using end-point values of each sim-ETG to predict each ERK feature. Dashed line represents the maximum value. **d** Linear regression using gene parameter values to predict how well each gene tracks with average ERK activity. The model uses the negative feedback rate, mRNA degradation rate, protein degradation rate, phosphorylated protein degradation rate, de-phosphorylation rate, and fraction baseline to predict the R^2 value from Fig. 7d. **e** Coefficient weights for linear regression in Fig. S10d. **f** Test set error (residual mean squared error) for each newly added gene in the prediction model.

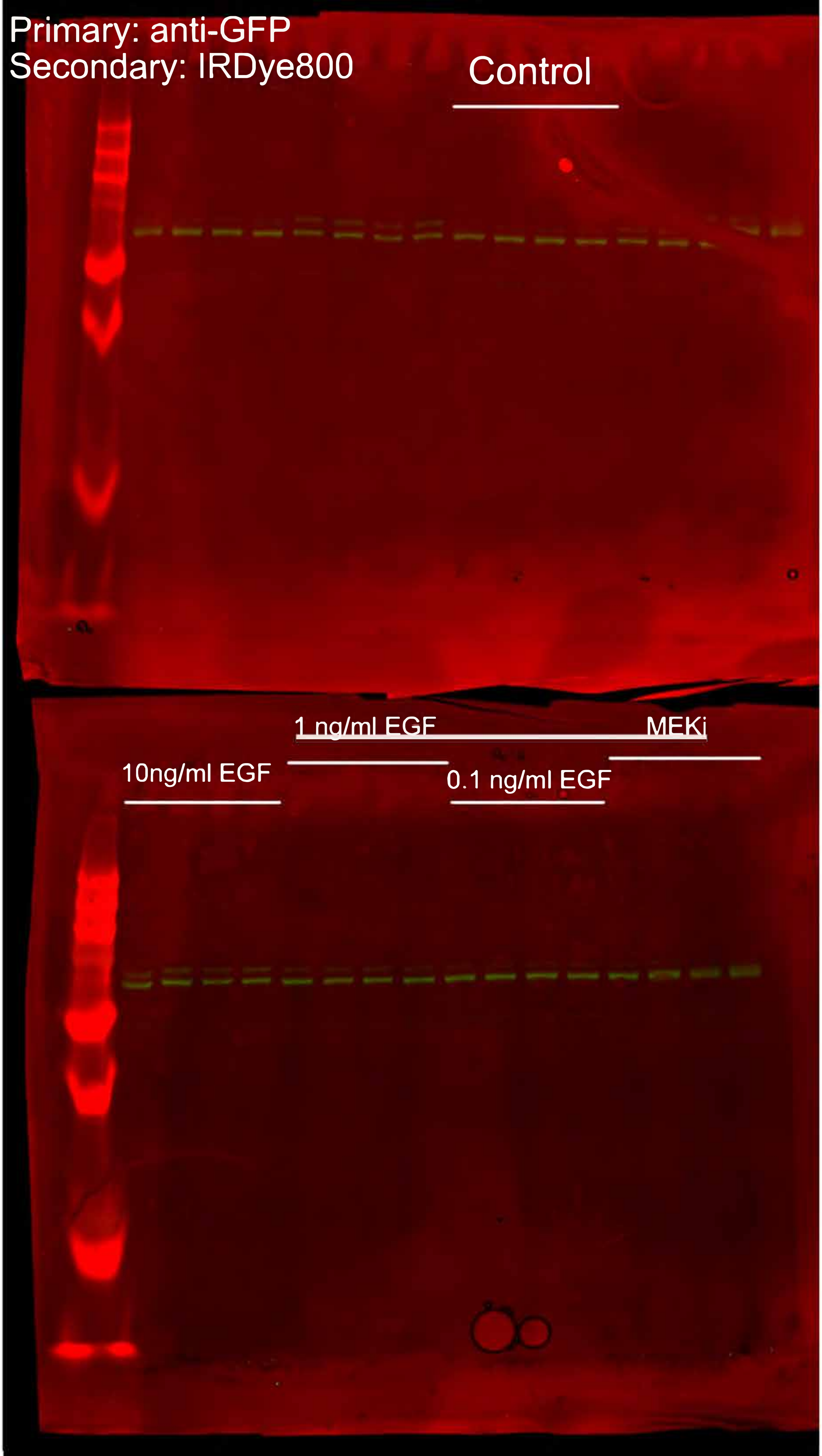
Primary: anti-GFP
Secondary: IRDye800
Control
1 ng/ml EGF
MEKi
10ng/ml EGF
0.1 ng/ml EGF

Supplementary Figure 11: Raw/uncropped image of Phos-Tag gels for EKAR3.5 calibration. Top: Gel #1 containing lysates treated with 0.1 ng/ml EGF, 10 ng/ml EGF, Imaging Media, or 1 ng/ml EGF (left to right, replicates of 4). Bottom: Gel #2 containing lysates treated with 0.1 ng/ml EGF, 10 ng/ml EGF, 1 ng/ml EGF or 1 100nM MEK inhibitor (left to right, replicates of 4). Primary antibody: Anti-Mouse GFP. Secondary: IRDye 800CW Donkey anti-Mouse IgG.

Supplementary table 1: List of treatments/conditions along with the number of replicate wells and replicate experiments.

Cell type	Treatment1	T1 Time (hr)	Treatment2	T2 Time (hr)	Well replicates	Experimental replicates (days)
MCF10A	EGF 0.01ng/ml	3			11	3
MCF10A	EGF 0.03164ng/ml	3			11	3
MCF10A	EGF 0.1ng/ml	3			9	3
MCF10A	EGF 0.3164ng/ml	3			10	3
MCF10A	EGF 1ng/ml	3			11	3
MCF10A	EGF 3.164ng/ml	3			11	3
MCF10A	EGF 10ng/ml	3			11	3
MCF10A	EGF 31.64ng/ml	3			11	3
MCF10A	PD 100nM	3			21	3
MCF10A	Imaging media control	3			32	3
MCF10A	EGF 0.01ng/ml	3	PD 100 nM	18	2	2
MCF10A	EGF 0.03164ng/ml	3	PD 100 nM	18	3	3
MCF10A	EGF 0.1ng/ml	3	PD 100 nM	18	3	3
MCF10A	EGF 0.3164ng/ml	3	PD 100 nM	18	3	3
MCF10A	EGF 1ng/ml	3	PD 100 nM	18	3	3
MCF10A	EGF 3.164ng/ml	3	PD 100 nM	18	3	3
MCF10A	EGF 10ng/ml	3	PD 100 nM	18	3	3
MCF10A	EGF 31.64ng/ml	3	PD 100 nM	18	3	3
MCF10A	EGF 0.01ng/ml	3	PD 100 nM	17	2	2
MCF10A	EGF 0.03164ng/ml	3	PD 100 nM	17	3	3
MCF10A	EGF 0.1ng/ml	3	PD 100 nM	17	3	3
MCF10A	EGF 0.3164ng/ml	3	PD 100 nM	17	3	3
MCF10A	EGF 1ng/ml	3	PD 100 nM	17	3	3
MCF10A	EGF 3.164ng/ml	3	PD 100 nM	17	3	3
MCF10A	EGF 10ng/ml	3	PD 100 nM	17	3	3
MCF10A	EGF 31.64ng/ml	3	PD 100 nM	17	3	3
MCF10A	EGF 0.01ng/ml	3	PD 100 nM	15	3	3
MCF10A	EGF 0.03164ng/ml	3	PD 100 nM	15	4	3
MCF10A	EGF 0.1ng/ml	3	PD 100 nM	15	4	3
MCF10A	EGF 0.3164ng/ml	3	PD 100 nM	15	4	3
MCF10A	EGF 1ng/ml	3	PD 100 nM	15	4	3
MCF10A	EGF 3.164ng/ml	3	PD 100 nM	15	4	3
MCF10A	EGF 10ng/ml	3	PD 100 nM	15	4	3
MCF10A	EGF 31.64ng/ml	3	PD 100 nM	15	4	3
MCF10A	Imaging media control	3	PD 100 nM	18	4	3
MCF10A	Imaging media control	3	PD 100 nM	17	2	1
MCF10A	Imaging media	3	PD 100 nM	15	2	1

	control					
MCF10A	EGF 31.64ng/ml	17.5			2	2
MCF10A	EGF 10ng/ml	17.5			1	1
MCF10A	EGF 0.01ng/ml	17.5			1	1
MCF10A	EGF 3.164ng/ml	17.5			1	1
MCF10A	EGF 0.01ng/ml	13			1	1
MCF10A	EGF 0.03164ng/ml	13			1	1
MCF10A	EGF 0.3164ng/ml	13			2	2
MCF10A	EGF 1ng/ml	13			4	2
MCF10A	EGF 3.164ng/ml	13			2	2
MCF10A	EGF 10ng/ml	13			2	2
MCF10A	EGF 31.64ng/ml	13			2	2
**Dataset2-Cancer Cell Tandem Plates:**						
Cell Type	Treatment1	T1 Time (hr)	Treatment2	T2 Time (hr)	Well Replicates	Experimental Replicates
A549	Imaging Media	3			3	3
A549	EGF10ng/ml	3			6	3
A549	EGF0.1ng/ml	3			6	3
A549	PD100nM	3			6	3
A549	EGF10ng/ml	3	PD 200nM	13	3	3
A549	EGF0.1ng/ml	3	PD 200nM	13	3	3
A549	Imaging Media	3	PD 200nM	13	3	3
A549	EGF10ng/ml	3	PD 200nM	17	3	3
A549	EGF0.1ng/ml	3	PD 200nM	17	3	3
A549	Imaging Media	3	PD 200nM	17	3	3
A549	EGF 10ng/ml	3		13	3	3
A549	EGF 0.1ng/ml	3		13	3	3
A549	Imaging Media	3		13	3	3
A549	EGF 10ng/ml	3		17.5	3	3
A549	EGF 0.1ng/ml	3		17.5	3	3
A549	Imaging Media	3		17.5	3	3
A549	Serum 10%	3		17.5	3	3
A549	Serum 10%	3		13	3	3
A549	Serum10%	3			3	3
A549	Serum10%PD	3	PD 200nM	17	3	3
A549	Serum10%PD	3	PD 200nM	13	3	3
HCC827	Imaging Media	3			3	3
HCC827	EGF10ng/ml	3			3	3
HCC827	EGF0.1ng/ml	3			6	3
HCC827	PD100nM	3			6	3
HCC827	EGF10ng/ml	3	PD 200nM	13	3	3
HCC827	EGF0.1ng/ml	3	PD 200nM	13	3	3
HCC827	Imaging Media	3	PD 200nM	13	3	3

HCC827	EGF10ng/ml	3	PD 200nM	17	3	3
HCC827	EGF0.1ng/ml	3	PD 200nM	17	3	3
HCC827	Imaging Media	3	PD 200nM	17	3	3
HCC827	EGF 10ng/ml	3		13	3	3
HCC827	EGF 0.1ng/ml	3		13	3	3
HCC827	Imaging Media	3		13	3	3
HCC827	EGF 10ng/ml	3		17.5	3	3
HCC827	EGF 0.1ng/ml	3		17.5	3	3
HCC827	Imaging Media	3		17.5	3	3
HCC827	Serum 10%	3		17.5	3	3
HCC827	Serum 10%	3		13	3	3
HCC827	Serum10%	3			3	3
HCC827	Serum10%PD	3		17	3	3
HCC827	Serum10%PD	3		13	3	3
MCF7	Imaging Media	3			3	3
MCF7	EGF10ng/ml	3			6	3
MCF7	EGF0.1ng/ml	3			6	3
MCF7	PD100nM	3			3	3
MCF7	EGF10ng/ml	3	PD 200nM	13	3	3
MCF7	EGF0.1ng/ml	3	PD 200nM	13	3	3
MCF7	Imaging Media	3	PD 200nM	13	3	3
MCF7	EGF10ng/ml	3	PD 200nM	17	3	3
MCF7	EGF0.1ng/ml	3	PD 200nM	17	3	3
MCF7	Imaging Media	3	PD 200nM	17	3	3
MCF7	EGF 10ng/ml	3		13	3	3
MCF7	EGF 0.1ng/ml	3		13	3	3
MCF7	Imaging Media	3		13	3	3
MCF7	EGF 10ng/ml	3		17.5	3	3
MCF7	EGF 0.1ng/ml	3		17.5	3	3
MCF7	Imaging Media	3		17.5	3	3
MCF7	Serum 10%	3		17.5	3	3
MCF7	Serum 10%	3		13	3	3
MCF7	Serum10%	3			3	3
MCF7	Serum10%PD	3	PD 200nM	17	3	2
MCF7	Serum10%PD	3	PD 200nM	13	3	2

Supplementary Table 2: List of gene parameters in the ordinary differential equation model

Parameter	Description	Unit	Value/Range
TF^T	Total transcription factor concentration	nM	1
k_{pTF}	ERK-dependent transcription factor phosphorylation rate	$nM^{-1}min^{-1}$	1e-1
k_{dTF}	Transcription factor de-phosphorylation rate	min^{-1}	5e-1;
k_b	Baseline target mRNA transcription rate	nM/min	1e-5
k_m	ERK-dependent target mRNA transcription rate	min^{-1}	1e-1
$k_{\emptyset m}$	Target mRNA degradation rate	min^{-1}	[1e-3, 1]
τ_m	Transcription delay	min	1
K_D	Negative feedback half-maximal concentration	nM	[1e-2, 100]
v	Feedback Hill Coef.	-	2
k_P	Target protein translation rate	min^{-1}	1e-1;
$k_{\emptyset P}$	Target protein degradation rate	min^{-1}	[1e-3, 1]
τ_P	Translation delay	min	10
k_{pP}	ERK-dependent target phosphorylation rate	$nM^{-1}min^{-1}$	1e-1
k_{dP}	Target protein de-phosphorylation rate	min^{-1}	[1e-3, 1]
$k_{\emptyset pP}$	Phosphorylated target degradation rate	min^{-1}	[1e-3, 1]

Supplementary Table 3: List of materials, software, and reagents used in the study.

Reagent or Resource	Source	Identifier/RRID
**Antibodies**		
Anti-Fra-1, clone C-12 Lot K0822 (1:200)	Santa Cruz Biotechnology	sc28310; AB_627632
Anti-c-Fos Lot GR3360368-1 (1:200)	abcam	ab190289; AB_2737414
Anti-cJun clone 60A8 Lot 13 (1:300)	Cell Signaling Technology	9165; AB_2130165
Anti-c-Myc clone D84C12 Lot 15 (1:500)	Cell Signaling Technology	5605; AB_1903938
Anti-DUSP1 Lot A119326 (1:400)	Sigma-Aldrich	HPA069577; NA
Anti-DUSP6 Lot 201-3G2 (1:40)	abnova	H00001848-M01; AB_489708
Anti-E-Cadherin Lot 8 (1:400)	Cell Signaling Technology	14472; AB_2728770
Anti-Egr-1, clone 44D5 Lot 4 (1:1600)	Cell Signaling Technology	4153; AB_2097035
Anti-EZH2 clone 144CT2.1.1.5 Lot YE3031422A (1:50)	ThermoFisher	MA5-18108; AB_2539482
Anti-FoxO1 clone D7C1H Lot 3 (1:200)	Cell Signaling Technology	14952; AB_2722487
Anti-GFP, clone 4B10 (1:1000)	Cell signaling	2955; AB_1196614
Anti-GSK-3β clone 3D10 Lot 5 (1:200)	Cell Signaling Technology	9832; NA
Anti-NF-1 Lot GR287164-3 (1:180)	abcam	178323; AB_2728814
Anti-Phospho-4E-BP1 (Thr37/46) clone 236B4 Lot 26 (1:200)	Cell Signaling Technology	2855; AB_560835
Anti-phospho-c-Fos clone D82C12 Lot 5 (1:200)	Cell Signaling Technology	5348; AB_10557109
Anti-Phospho-EGF Receptor (Tyr1068) cloneD7A Lot 17 (1:200)	Cell Signaling Technology	3777; AB_2096270
Anti-phospho-ERK (p44/42) clone D13.14.4E Lot 28 (1:200)	Cell Signaling Technology	4370; AB_2315112
Anti-phospho-Rb (Ser807/811) clone D20B12 Lot 8 (1:1600)	Cell Signaling Technology	8516; AB_11178658
Anti-Phospho-S6 Ribosomal Protein (Ser235/236) clone D57.2.2E Lot 16 (1:200)	Cell Signaling Technology	4858; AB_916156
Anti-Rsk1 clone 964203 Lot CKPN0116121 (1:60)	R&D Systems	MAB992; NA

Donkey anti-Rabbit IgG (H+L) Alexa Fluor 555 Lot 2339822 (1:500)	ThermoFisher	A-31572; AB_162543
Goat anti-Mouse IgG (H+L) Alexa Fluor 647 Lot 2229182 (1:500)	ThermoFisher	A-21236; AB_2535805
IRDye 800CW Donkey anti-Mouse IgG (1:10000)	Licor	926-32212; AB_621847
**Chemicals, Peptides, and Recombinant Proteins**		
Epidermal growth factor	Peprotech	AF-100-15
0.25% Trypsin-EDTA	Life Technologies	25200-056
Ammonium chloride	Sigma-Aldrich	254134
Bovine Serum Albumin	Sigma-Aldrich	A7906
Bromophenol blue	Sigma-Aldrich	B5525
Cholera Toxin	Sigma-Aldrich	C8052
Collagen I, rat tail	Life Technologies	A10483-01
Dithiothreitol	Fisher	BP172
DMEM	Gibco	11965-092
DMEM/F-12 1:1	Life Technologies	11320
Fetal Bovine Serum	GeminiBio	100-106-500
Glycine	Fisher	BP381-500
Glycine (Crystalline Powder)	Fisher	BP381
Guanidinum Hydrochloride,	Fisher	BP178-500
Halt Protease inhibitor cocktail	ThermoFisher	1861278
Heat Inactivated Horse Serum	Life Technologies	26050
Hoechst-33342	Life Technologies	H3570
Hydrocortisone	Sigma-Aldrich	H0888
Insulin	Sigma-Aldrich	I9278
l-Glutamine	Life Technologies	25030-081
Maleimide	Sigma-Aldrich	129585
N-Acetyl-L-cysteine	Sigma-Aldrich	A7250
Neomycin	Sigma-Aldrich	N6386
Odyssey Blocking Buffer (PBS)	Licor	927-40000
Paraformaldehyde	ThermoFisher	043368.9M

PD0325901	Selleck Biochemicals	S1036
Penicillin streptomycin	Life Technologies	15070-063
Phosphate-buffered saline,	Fisher	BP399-1
Ponceau S solution, suitable for electrophoresis, 0.1% (*w*/*v*) in 5% acetic acid, 1L	Sigma-Aldrich	P7170-1L
RPMI-1640	Sigma	R0883
TCEP hydrochloride	ApexBio	B6055
Tris Base	Fisher	BP152
Tween-20	Fisher	BP337100
Urea	Fisher	U15-500
**Experimental Models/Cell Lines**		
Human: MCF-10A, clone 5E	Joan Brugge, Harvard Medical School	RRID:CVCL_0598
A549	ATCC	CRM-CCL-185
HCC827	ATCC	CRL-2868
MCF7	ATCC	HTB-22
**Recombinant DNA**		
Plasmid: pPBJ-EKAR3.5nls-neo	Cloned from Sparta et al. 2015	To be deposited on addgene
**Software and Algorithms**		
NIS-Elements AR ver. 4.20	Nikon	RRID:SCR_014329
Bio-Formats ver. 5.1.1 (May 2015)	OME	RRID:SCR_000450
Gramm Visualization Toolbox	Pierre Morel	https://www.mathworks.com/matlabcentral/fileexchange/54465-gramm-data-visualization-toolbox
ImageJ (1.52p)	National Institutes of Health	RRID:SCR_003070
MATLAB 2020a	Mathworks	SCR_001622
Python (3.8.16)	Python Software Foundation	RRID:SCR_008394
Turbo colormap	Google Rearch	https://research.google/blog/turbo-an-improved-rainbow-colormap-for-visualization/
uTrack 2.0	(Jaqaman et al., 2008)	http://www.utsouthwestern.edu/labs/danuser/software/
**Proofreading software**		
Grammarly	Grammarly, Inc	www.grammarly.com/
ChatGPT	OpenAI	www.openai.com

Other		
Glass Bottom Plates, #1.5 cover glass	In Vitro Scientific	P24-1.5H-N, P96-1.5H-N
SuperSep Phos-tag gels (50 μmol/l), 12.5%, 17 wells	Wako-Chem	195-17991

Supplementary Table 4: p-values associated with Figure 6b.

	Group A	Group B	p-value
pERK	1	2	0.9759
	1	3	0.0108
	1	4	0.9701
	1	5	0.0038
	2	3	0.0046
	2	4	1
	2	5	0.0017
	3	4	0.0044
	3	5	0.9463
	4	5	0.0016
Fra-1	1	2	0.6344
	1	3	0.9934
	1	4	0.3561
	1	5	0.0119
	2	3	0.4153
	2	4	0.9818
	2	5	0.0016
	3	4	0.2068
	3	5	0.0221
	4	5	7.78E-04
c-Myc	1	2	0.546
	1	3	1.52E-06

	1	4	0.3903
	1	5	2.74E-05
	2	3	4.95E-07
	2	4	0.9979
	2	5	6.50E-06
	3	4	4.05E-07
	3	5	0.044
	4	5	5.04E-06
pc-Fos	1	2	0.9913
	1	3	1.05E-04
	1	4	0.9797
	1	5	0.0011
	2	3	6.58E-05
	2	4	1
	2	5	6.12E-04
	3	4	5.86E-05
	3	5	0.3622
	4	5	5.32E-04
c-Fos	1	2	0.9125
	1	3	0.0615
	1	4	0.8611
	1	5	0.0035
	2	3	0.0168
	2	4	0.9999
	2	5	0.0011
	3	4	0.0137
	3	5	0.3761
	4	5	9.29E-04
c-Jun	1	2	0.9827

	1	3	0.9839
	1	4	0.6997
	1	5	1
	2	3	0.832
	2	4	0.9335
	2	5	0.9821
	3	4	0.419
	3	5	0.9845
	4	5	0.6972
Egr-1	1	2	0.9807
	1	3	0.4077
	1	4	0.9844
	1	5	0.0675
	2	3	0.1997
	2	4	1
	2	5	0.0292
	3	4	0.2086
	3	5	0.7169
	4	5	0.0306
pRb	1	2	0.0374
	1	3	0.1716
	1	4	0.0361
	1	5	0.0075
	2	3	0.001
	2	4	1
	2	5	8.89E-05
	3	4	9.93E-04
	3	5	0.3113
	4	5	8.67E-05
