# Supplementary material for: Deciphering the history of ERK activity from fixed-cell immunofluorescence measurements
Source: Nat Commun. 2025 May 21;16:4721. doi: 10.1038/s41467-025-58348-7 (PMC12095524; doi:10.1038/s41467-025-58348-7)
Supplement: Supplementary file 2 — Reporting Summary [file 41467_2025_58348_MOESM2_ESM.pdf]

nature portfolio

Corresponding author(s): John Albeck
Last updated by author(s): Feb 4, 2025

Reporting Summary

Nature Portfolio wishes to improve the reproducibility of the work that we publish. This form provides structure for consistency and transparency in reporting. For further information on Nature Portfolio policies, see our Editorial Policies and the Editorial Policy Checklist.

Statistics

For all statistical analyses, confirm that the following items are present in the figure legend, table legend, main text, or Methods section.

n/a	Confirmed	
☐	☒	The exact sample size (*n*) for each experimental group/condition, given as a discrete number and unit of measurement
☐	☒	A statement on whether measurements were taken from distinct samples or whether the same sample was measured repeatedly
☐	☒	The statistical test(s) used AND whether they are one- or two-sided<br>*Only common tests should be described solely by name; describe more complex techniques in the Methods section.*
☒	☐	A description of all covariates tested
☒	☐	A description of any assumptions or corrections, such as tests of normality and adjustment for multiple comparisons
☐	☒	A full description of the statistical parameters including central tendency (e.g. means) or other basic estimates (e.g. regression coefficient) AND variation (e.g. standard deviation) or associated estimates of uncertainty (e.g. confidence intervals)
☐	☒	For null hypothesis testing, the test statistic (e.g. *F*, *t*, *r*) with confidence intervals, effect sizes, degrees of freedom and *P* value noted<br>*Give P values as exact values whenever suitable.*
☒	☐	For Bayesian analysis, information on the choice of priors and Markov chain Monte Carlo settings
☒	☐	For hierarchical and complex designs, identification of the appropriate level for tests and full reporting of outcomes
☐	☒	Estimates of effect sizes (e.g. Cohen's *d*, Pearson's *r*), indicating how they were calculated

Our web collection on statistics for biologists contains articles on many of the points above.

Software and code

Policy information about availability of computer code

Data collection	Data were collected using Nikon Elements Software
Data analysis	Data analyses were performed in MATLAB and Python; all code has been linked as a a figshare repository

For manuscripts utilizing custom algorithms or software that are central to the research but not yet described in published literature, software must be made available to editors and reviewers. We strongly encourage code deposition in a community repository (e.g. GitHub). See the Nature Portfolio guidelines for submitting code & software for further information.

Data

Policy information about availability of data

All manuscripts must include a data availability statement. This statement should provide the following information, where applicable:

- Accession codes, unique identifiers, or web links for publicly available datasets
- A description of any restrictions on data availability
- For clinical datasets or third party data, please ensure that the statement adheres to our policy

All source data, which include processed cell intensity values, have been linked to the manuscript as a figshare repository

Research involving human participants, their data, or biological material

Policy information about studies with human participants or human data. See also policy information about sex, gender (identity/presentation), and sexual orientation and race, ethnicity and racism.

Reporting on sex and gender	The cell lines MCF10A, MCF7, and HCC827 were derived from female humans. The cell line A549 was derived from a male human.
Reporting on race, ethnicity, or other socially relevant groupings	Not applicable
Population characteristics	Not applicable
Recruitment	Not applicable
Ethics oversight	Not applicable

Note that full information on the approval of the study protocol must also be provided in the manuscript.

Field-specific reporting

Please select the one below that is the best fit for your research. If you are not sure, read the appropriate sections before making your selection.

[x] Life sciences [ ] Behavioural & social sciences [ ] Ecological, evolutionary & environmental sciences

For a reference copy of the document with all sections, see nature.com/documents/nr-reporting-summary-flat.pdf

Life sciences study design

All studies must disclose on these points even when the disclosure is negative.

Sample size	We collected the data on the maximum number of cells feasible within the the timeframe of the study. Ultimately, live-cell and immunofluorescence data were collected for over 90,000 cells, which represents, to our knowledge, the largest such dataset reported.
Data exclusions	Following automated processing to identify and track cells, cells that were tracked for fewer than 150 timepoints were omitted from subsequent analysis. Cells that had immunofluorescence intensity values below background were also omitted.
Replication	Data were collected in three separate replicate experiments. Batch effects in fluorescence intensity, which are expected from run to run, were examined and corrected for. No other significant variation was noted between the experimental runs.
Randomization	In our experimental setup, all cells plated were from the same parental population, and the number of cells plated and subjected to each condition (>10,000 cells/well) was large enough to be considered representative of the parental population. Comparison of replicate wells showed no significant variation in their distributions, confirming this assumption.
Blinding	Cell segmentation, tracking, and analysis were fully automated and did not involve human input.

Reporting for specific materials, systems and methods

We require information from authors about some types of materials, experimental systems and methods used in many studies. Here, indicate whether each material, system or method listed is relevant to your study. If you are not sure if a list item applies to your research, read the appropriate section before selecting a response.

Materials & experimental systems

n/a	Involved in the study	
[ ]	[x]	Antibodies
[ ]	[x]	Eukaryotic cell lines
[x]	[ ]	Palaeontology and archaeology
[x]	[ ]	Animals and other organisms
[x]	[ ]	Clinical data
[x]	[ ]	Dual use research of concern
[x]	[ ]	Plants

Methods

n/a	Involved in the study	
[x]	[ ]	ChIP-seq
[x]	[ ]	Flow cytometry
[x]	[ ]	MRI-based neuroimaging

Antibodies

Antibodies used	Anti-Fra-1, 1:200, clone C-12 Santa Cruz Biotechnology sc28310; AB_627632

	Anti-c-Fos, 1:200, abcam ab190289; AB_2737414<br>Anti-cJun, 1:300, clone 60A8, Cell Signaling Technology 9165; AB_2130165<br>Anti-c-Myc, 1:500, clone D84C12, Cell Signaling Technology 5605; AB_1903938<br>Anti-DUSP1, 1:400, Sigma-Aldrich HPA069577; NA<br>Anti-DUSP6, 1:400, abnova H00001848-M01; AB_489708<br>Anti-E-Cadherin, 1:400, Cell Signaling Technology 14472; AB_2728770<br>Anti-Egr-1, 1:1600, clone 44D5, Cell Signaling Technology 4154; AB_2097035<br>Anti-EZH2, 1:50, clone 144CT2.1.1.5, ThermoFisher MA5-18108; AB_2539482<br>Anti-FoxO1, 1:200, clone D7C1H, Cell Signaling Technology 14952; AB_2722487<br>Anti-GFP, 1:1000, clone 4B10, Cell signaling 2955; AB_1196614<br>Anti-GSK-3β, 1:200, clone 3D10, Cell Signaling Technology 9832; NA<br>Anti-NF-1, 1:180, abcam 178323; AB_2728814<br>Anti-Phospho-4E-BP1, 1:200, (Thr37/46) clone 236B4, Cell Signaling Technology 2855; AB_560835<br>Anti-phospho-c-Fos, 1:200, clone D82C12, Cell Signaling Technology 5348; AB_10557109<br>Anti-Phospho-EGFR Receptor (Tyr1068), 1:200, cloneD7A, Cell Signaling Technology 3777; AB_2096270<br>Anti-phospho-ERK (p44/42), 1:200, clone D13.14.4E, Cell Signaling Technology 4370; AB_2315112<br>Anti-phospho-Rb (Ser807/811), 1:1600, clone D20B12, Cell Signaling Technology 8516; AB_11178658<br>Anti-Phospho-S6 Ribosomal Protein (Ser235/236), 1:200, clone D57.2.2E, Cell Signaling Technology 4858; AB_916156<br>Anti-Rsk1, 1:60, clone 964203, R&D Systems MAB992; NA<br>Donkey anti-Rabbit IgG (H+L) Alexa Fluor 555, 1:500, ThermoFisher A-31572; AB_162543<br>Goat anti-Mouse IgG (H+L) Alexa Fluor 555, 1:500, ThermoFisher A-21235; NA<br>IRDye 800CW Donkey anti-Mouse IgG, 1:10000, Licor 926-32212; AB_621847
Validation	All antibodies used were validated by manufacturers for use in immunofluorescence experiments. We confirmed the function of each antibody in our immunofluorescence application by 1) appropriate intracellular localization, and 2) expected change in fluorescence upon growth factor stimulation, 3) expected loss or change in fluorescence following EGF stimulation or MEK inhibition.

Eukaryotic cell lines

Policy information about cell lines and Sex and Gender in Research

Cell line source(s)	MCF10A cells were received directly from stocks maintained and extensively characterized by Joan Brugge's lab at Harvard Medical School. A549, MCF7, and HCC827 cells were received from the American Type Culture Collection (ATCC)
Authentication	Given the sources of the cell line, genomic authentication tests were deemed unnecessary and were not performed.
Mycoplasma contamination	Cell lines were tested for mycoplasma and found to be negative
Commonly misidentified lines (See ICLAC register)	Not applicable

Plants

Seed stocks	Not applicable
Novel plant genotypes	Not applicable
Authentication	Not applicable
