# Supplementary material for: Deciphering the history of ERK activity from fixed-cell immunofluorescence measurements
Source: Nat Commun. 2025 May 21;16:4721. doi: 10.1038/s41467-025-58348-7 (PMC12095524; doi:10.1038/s41467-025-58348-7)
Supplement: Supplementary file 3 — Description of Additional Supplementary Files [file 41467_2025_58348_MOESM3_ESM.docx]

**Deciphering the History of ERK Activity from Fixed-Cell Immunofluorescence Measurements**

Abhineet Ram^1^, Michael Pargett^1^, Yongin Choi^1^, Devan Murphy^1^, Carolyn Teragawa^1^, Markhus Cabel^1^, Nont Kosaisawe^1^, Gerald Quon^1^, John G. Albeck^1,^*

Additional supplementary files:

**Supplementary Movie 1**: Time-lapse imaging of MCF10A cells expressing EKAR3.1 followed by immunofluorescence measurements. Live imaging: gray/light orange coloring of cells represents low ERK activation whereas dark orange/red represents high ERK activation. Cells were treated with 1ng/ml EGF at 3 hr.

**Supplementary Movie 2**: Time-lapse imaging of HCC827 cells expressing EKAR3.1 followed by immunofluorescence (IF) measurements. Live imaging: gray/light orange coloring of cells represents low ERK activation whereas dark orange/red represents high ERK activation. Cells were treated with 0.1ng/ml EGF at 3 hr. HCC827, A549, and MCF7 cells were imaged and stained together on the sample plate, therefore EKAR3.1 and IF intensities are comparable with Supplementary Movies 3 and 4.

**Supplementary Movie 3**: Time-lapse imaging of A459 cells expressing EKAR3.1 followed by immunofluorescence measurements. Live imaging: gray/light orange coloring of cells represents low ERK activation whereas dark orange/red represents high ERK activation. Cells were treated with 0.1ng/ml EGF at 3 hr.

**Supplementary Movie 4**: Time-lapse imaging of MCF7 cells expressing EKAR3.1 followed by immunofluorescence measurements. Live imaging: gray/light orange coloring of cells represents low ERK activation whereas dark orange/red represents high ERK activation. Cells were treated with 0.1ng/ml EGF at 3 hr.
